# Avian influenza in birds: Insights from a comprehensive review

**DOI:** 10.14202/vetworld.2024.2544-2555

**Published:** 2024-11-13

**Authors:** Siti Rani Ayuti, Aswin Rafif Khairullah, Mirni Lamid, Mohammad Anam Al-Arif, Sunaryo Hadi Warsito, Otto Sahat Martua Silaen, Ikechukwu Benjamin Moses, Intan Permatasari Hermawan, Sheila Marty Yanestria, Mira Delima, Teuku Reza Ferasyi, Suhita Aryaloka

**Affiliations:** 1 Doctoral Program of Veterinary Science, Faculty of Veterinary Medicine, Universitas Airlangga, Surabaya, East Java, Indonesia; 2 Laboratory of Biochemistry, Faculty of Veterinary Medicine, Universitas Syiah Kuala, Banda Aceh, Aceh, Indonesia; 3 Research Center for Veterinary Science, National Research and Innovation Agency, Bogor, West Java, Indonesia; 4 Division of Animal Husbandry, Faculty of Veterinary Medicine, Universitas Airlangga, Surabaya, East Java, Indonesia; 5 Doctoral Program in Biomedical Science, Faculty of Medicine, Universitas Indonesia, Jakarta, Indonesia; 6 Department of Applied Microbiology, Faculty of Science, Ebonyi State University, Abakaliki, Nigeria; 7 Laboratory of Internal Medicine, Faculty of Veterinary Medicine, Universitas Wijaya Kusuma Surabaya, Surabaya, East Java, Indonesia; 8 Laboratory of Veterinary Public Health, Faculty of Veterinary Medicine, Universitas Wijaya Kusuma Surabaya, Surabaya, East Java, Indonesia; 9 Department of Animal Husbandry, Faculty of Agriculture, Universitas Syiah Kuala, Banda Aceh, Indonesia; 10 Laboratory of Veterinary Public Health, Faculty of Veterinary Medicine, Universitas Syiah Kuala, Banda Aceh, Indonesia; 11 Center for Tropical Veterinary Studies, One Health Collaboration Center, Universitas Syiah Kuala, Banda Aceh, Aceh, Indonesia; 12 Master Program of Veterinary Agribusiness, Faculty of Veterinary Medicine, Universitas Airlangga, Surabaya, East Java, Indonesia

**Keywords:** avian influenza, disease, human health, poultry, virus

## Abstract

One of the worst zoonotic illnesses, avian influenza (AI), or commonly referred to as bird flu, is caused by viruses belonging to the genus Influenza viruses, which are members of the Orthomyxoviridae family. The harmful effects of AI illness can affect both human and animal health and cause financial losses. Globally, the AI virus lacks political purpose and is not limited by geographical limits. It has been isolated from poultry, wild birds, and captive birds in Asia, North America, Europe, Australia, and South America. Their virulence is divided into highly pathogenic AI (HPAI) and low pathogenic AI (LPAI). The AI virus can also be diagnosed in a laboratory setting using molecular tests like real-time polymerase chain reaction or serological tests like the hemagglutinin inhibition test, agar gel immunodiffusion, antigen detection enzyme-linked immunosorbent assay, and other immunoassays. The type of AI virus and host species determines the clinical manifestations, severity, and fatality rates of AI. Human infection with AI viruses typically results from direct transmission from infected birds to humans. AI outbreaks in domestic and wild birds are uncommon; however, an infection can pose a significant threat to public, veterinary, and medical health. Successful vaccination reduces the probability of AI H5N1 virus infection in meat and other poultry products and prevents systemic infection in chickens. This review will provide information that can be used as a reference for recognizing the dangers of AI and for preventing and controlling the disease, considering its potential to become a serious pandemic outbreak.

## Introduction

The rapid spread of infectious diseases substantially influences poultry productivity [1]. Diseases such as avian influenza (AI), Marek’s disease, infectious bursal disease, and other respiratory illnesses can rapidly and readily spread among poultry housed in production systems [2]. Poultry production is a hazardous endeavor due to fatal strains of infectious agents, which further restrict the poultry industry’s growth within the nation’s economy [3]. In recent years, the global health community has directed its attention toward the increasing prevalence of AI transmission [4]. As the number of AI cases increases, the illness begins to be seen as a potentially infectious pandemic threat [5].

One of the worst zoonotic illnesses, AI, commonly referred to as bird flu, is caused by viruses belonging to the genus Influenza viruses, which are members of the *Orthomyxoviridae* family [6]. These viruses have a genome of eight single-stranded negative-sense RNA segments [7]. The AI virus has two subtypes based on glycoproteins, namely, Neuraminidase (NA) and Hemagglutinin (HA) on its surface, which, in addition to its infectivity, are the primary factors influencing the AI virus’ pathogenicity, transmission, and host adaptation [8]. Although this virus primarily affects poultry, it can also infect humans, pets, livestock, and wild animals [9].

The harmful effects of AI illness can affect both human and animal health and cause financial losses [10]. In humans, AI is classified as a highly contagious respiratory illness that is usually self-limiting but has a significant global impact on morbidity and mortality [5]. In poultry, severe pathogenicity can result in death, but it often has low pathogenicity, causing subclinical infections, respiratory conditions, or decreased egg production [11]. The clinical symptoms of this disease are difficult to detect because they are similar to those of other poultry diseases, for example, a decrease in egg production, which is a clinical symptom of fowl cholera, Newcastle disease (ND), infectious laryngotracheitis (ILT), infectious bronchitis (IB), and *Escherichia coli* infection [9].

The spread of AI was initially limited to Southeast Asia, but this virus has now migrated to Europe, the Middle East, and countries in the former Soviet Union [12]. The AI virus naturally inhabits wild water birds [13]. Typically, infection only results in clinical symptoms when the AI virus and its host coexist perfectly [14]. In addition, the annual global migration of wild birds spreads the virus worldwide, increasing its contagiousness [15]. The fact that vaccination and concurrent infection with low pathogenic diseases can prevent infected birds from displaying symptoms of illness or death but do not prevent birds from contracting highly pathogenic AI (HPAI) viruses are significant but frequently disregarded factors in the analysis of AI disease risks [16].

Poultry, particularly chickens and ducks, was the source of the AI outbreak, ultimately connected to human transmission [17]. AI illness is difficult to control because people regularly come into contact with chickens, ducks, birds, turkeys, and other poultry in daily life, like at farms, marketplaces, and slaughterhouses [5]. Since there is currently no effective treatment for AI virus infections in commercial poultry and no widely available vaccine for human AI, treatment options for human infections are limited to supportive therapy and antiviral medication. Resistance to antivirals is becoming a more significant issue [18].

This review aimed to explain the etiology, history, epidemiology, pathogenesis, diagnosis, clinical symptoms, transmission, risk factors, public health importance, economic impact, treatment, vaccination, and control of AI. This review will provide information that can be used as a reference for recognizing the dangers of AI and for preventing and controlling the disease, considering its potential to become a serious pandemic outbreak.

## Etiology

The RNA virus, termed the AI virus, is a member of the *Orthomyxoviridae* family [19]. This virus has a single-stranded nucleic acid composed of eight gene segments that encode approximately 11 proteins [20]. The influenza virus envelope comprises a combination of proteins and carbohydrates [7]. The virus uses its spikes to cling to particular receptors in host cells [21]. There are two types of spikes, namely, those containing NA and HA, which are situated outside the virion [22]. The four types of antigens found in influenza viruses are nucleocapsid protein (NP), HA, matrix protein (MP), and NA [23].

Based on the types of NP and MP antigens, influenza viruses are classified as influenza A, B, and C viruses [24]. Influenza A virus infection is highly harmful to both humans and animals, resulting in high rates of morbidity and mortality worldwide and making it a crucial component of the health sector [5]. Because this type of virus is easily mutable and can produce new, more virulent forms through antigenic drift or shift, it can spread globally [25]. There are nine NA and 15 HA subtypes [7].

Epidemiological seroprevalence investigations have demonstrated that a number of influenza A virus subtypes, including H2N2 (1889), H3N8 (1900), H1N1 (1918), H2N2 (1957), H3N2 (1968), H7N7 (1977), and H5N1 (2005), are linked to pandemic outbreaks [26]. Although influenza C virus is infrequently encountered despite its ability to infect both humans and animals, influenza B virus exclusively targets humans [27]. Types B and C influenza viruses infrequently or never produce pandemic outbreaks.

## History

The most frequently given date for the historical onset of AI, formerly the avian plague, was 1878, when the illness was identified as distinct from other illnesses that resulted in significant bird fatality rates [28]. Until 1880, the illness was an acute septicemic type of avian cholera [29]. A filterable agent caused the disease in 1901, but the virus was not recognized as an influenza virus until 1955 [30]. However, outbreaks of poultry diseases, such as ND, continued into the 1950s [31].

There were 15 epidemics of the AI virus in chickens between 1959 and 1995; however, the losses were minimal [28]. However, there have been at least 11 AI epidemics in poultry between 1996 and 2008, four of which involved millions of chickens [32]. Before the 1990s, AI in chickens resulted in a significant death rate; nonetheless, infections were rare and treatable [33]. The first report of human infection in Hong Kong was recorded in 1997 [34]. Since 2003, there have been over 700 documented incidences of Asian H5N1 AI in humans, and over 60 countries have been affected by these incidents, which have primarily occurred in 15 different Middle Eastern, European, African, and Asian countries [35].

## Epidemiology

Globally, the AI virus lacks political purpose and is not limited by geographical limits [36]. It has been isolated from captive, poultry, and wild birds in Asia, North America, Europe, South America, and Australia [37]. An AI anti-virus has also been found in Antarctic penguins, indicating that AI anti-virus medication is used to treat AI [38]. For example, the number of infected animals and the broader geographic dispersion are significant characteristics of outbreaks of highly virulent AI virus infections throughout Asia, Europe, Africa, and the Middle East [4]. As evidenced by epidemics of migratory birds in isolated regions like Mongolia, migrating birds may have a significant role in the geographic transmission of AI viruses, even though human activity may also play a role [13].

AI viruses are widespread in wild birds, although specific viruses vary by location [39]. In temperate regions of the world, annual influenza epidemics occur regularly and exhibit an amazing seasonal pattern, with peak incidence during the colder months of the year [40]. These yearly outbreaks vary in intensity. Recurrent AI outbreaks in Africa are caused by migratory birds’ flyways, which connect endemic and recently affected countries with free territories around the world, and the risk of transmission through legal and criminal trade [41]. Ethiopia and Kenya, two Sub-Saharan nations, have not seen any outbreak of the virus; nonetheless, it has been spreading to nearby nations like Sudan, where the bird can enter through a number of unofficial channels, including illegal bird trade [42]. Nonetheless, multiple outbreaks of this illness have been reported in Ghana [43] and Nigeria [44].

The World Health Organization (WHO) [45] reported that as of March 31, 2022, there have been 239 cases of human infection with the AI virus in the Western Pacific region. This represents the epidemiology of AI since January 2003. There were 134 fatal cases, indicating that the case fatality rate was 56% [46]. The first reports of AI virus infections in Indonesia were made in 2003 for birds and in 2005 for humans [10]. In August 2015, 844 cases of AI virus infection were reported, with 449 deaths. Most cases occurred in East Asia, and several were found in Eastern Europe and North Africa [47]. Based on the WHO update for the West Pacific region as of March 31, 2022, the H5N1 subtype AI has a high fatality rate of 56%, whereas the H7N9 virus has a mortality rate of 39% [45].

## Pathogenesis

AI viruses can infect and kill various bird species. Their virulence divides them into two categories. First, an HPAI virus termed HPAI has been identified as a lethal virus-producing bird plague [48]. This group was restricted to H5 and H7 rats, and the mortality rate was approximately 100% [49]. Second, another virus known as low pathogenic AI (LPAI) causes mild respiratory sickness [50]. There is uncertainty regarding the factors leading to the virus’s transformation from LPAI to HPAI. Under certain conditions, mutations occur quickly once wild birds are introduced. In other instances, the LPAI virus was present in chickens for months before mutation [51].

The pathogenicity of AI viruses is polygenic and heavily dependent on a group of genes that affect immune evasion mechanisms, replication efficiency, and host and tissue tropism [52]. Furthermore, after interspecies transmission, variables specific to the host and species affect the course of infection. There are several ways in which the LPAI virus can infect flocks of chickens [50]. These viruses can evolve into extremely harmful forms in susceptible poultry populations [32]. The inhalation or consumption of infectious LPAI or HPAI virions triggers pathogenesis because trypsin-like enzymes in intestinal and respiratory epithelial cells cleave surface HA [53]. This leads to multiple replication cycles in the intestines and respiratory tract, which release infectious virions [54].

Second, HPAI viruses infiltrate the submucosa and enter the capillaries following their initial replication in the respiratory epithelium [55]. This virus reproduces in endothelial cells and then travels through lymphatic and vascular networks to infect and multiply in different cell types in the skin, brain, and visceral organs [56]. Alternatively, the virus could spread throughout the body before multiplying extensively in vascular endothelial cells [57]. This virus is present in red, white, and plasma blood cells [5]. Macrophages appear to be involved in viral dissemination throughout the body [58]. This pantropic replication is caused by HA proteolytic cleavage sites, which are cleaved by the ubiquitous cellular enzyme furin [59]. Multiple organ failures lead to clinical symptoms and mortality.

Third, the intestine or respiratory tract is typically the only place where LPAI viruses can replicate [7]. Most frequently, respiratory injury results in the onset of disease or death, particularly when coupled with subsequent bacterial infection [60]. The LPAI virus replicates and damages renal tubules, pancreatic acinar epithelium, fallopian tubes, and other organs containing epithelial cells that occasionally have trypsin-like enzymes in several animals [61]. There is limited knowledge of the pathogenesis of AI virus infection in non-gallinaceous birds.

## Diagnosis

AI viruses cannot be identified based only on clinical signs and symptoms because the lesions and symptoms of this illness are diverse and may be mistaken for those of other illnesses. As a result, this disease cannot be clinically distinguished from other diseases such as ND, IB, fowl cholera, ILT, and *E. coli* infection [62]. Therefore, serological and virological testing is required, and confirmation must be performed at a qualified laboratory.

Oropharyngeal, cloacal, and tracheal swabs from live birds can be used to detect the AI virus [63]. Several factors, including the virus and bird species, affect the accuracy of this detection. Very tiny swabs might be helpful for small birds, but if cloacal sampling is not feasible (i.e., it cannot be gathered without harming the bird), droppings could be used instead [64]. Moreover, feathers from young birds can be used as helpful samples [65]. Samples of internal organs from deceased birds thought to have AI were also examined [66].

All species can benefit from virus characterization through virus isolation, which involves inoculating samples into chicken embryos to identify the characteristics of red blood cell deposition [67]. Despite being time-consuming, this method is the “gold standard” for detecting AI viruses and is mostly utilized for the diagnosis of initial clinical cases as well as the isolation of the virus for additional laboratory investigations [68]. The AI virus can also be diagnosed in a laboratory setting using molecular tests such as real-time polymerase chain reaction (RT-PCR) or serological tests such as the HA inhibition test, antigen detection enzyme-linked immunosorbent assay (ELISA), agar gel immunodiffusion (AGID), and other immunoassays [69–72].

The virus can be identified as influenza A virus through the hemagglutination inhibition test, in which the HA protein from AI can agglutinate erythrocytes from a number of species, including horses [73]. The hemagglutination response is inhibited or prevented by antibodies that target the antigenic regions of the AI HA molecule [74]. Therefore, when a standard AI antigen is provided as a reference material, a hemagglutination inhibition test can be performed to assess a patient’s antibodies to the AI virus.

The presence of the AI virus in amnio-allantoic and chorio-allantoic fluid can also be satisfactorily demonstrated by the agar gel immunodiffusion assay (AGID) test using nucleocapsid or matrix antigens; however, solid-phase antigen-capture ELISAs are a useful alternative for expedited and commercial studies [72, 75]. This method detects AI viruses using monoclonal antibodies specific to nucleoproteins [75]. The primary benefit of this test is its ability to detect the AI virus in as little as 15 min. This approach has several drawbacks, including less sensitivity, a possible lack of validation for various bird species, an inability to identify subtypes, and high equipment costs [76].

Another effective method for determining the genome of an AI virus is RT-PCR, which enables sensitive and targeted detection of viral nucleic acids [77]. The RT-PCR of clinical specimens with correct primer determination can rapidly detect and identify subtypes (at least H5 and H7), including DNA products that can be used for nucleotide sequencing [78]. However, RT-PCR is the recommended molecular detection technique for AI viruses; a variation in RT-PCR can speed up the process of identifying the virus subtype and sequencing it [79]. The vulnerability of RT-PCR is its susceptibility to contamination and the possibility of false-positive results [80].

The following illnesses need to be considered when making an AI differential diagnosis because they can cause rapid disease onset, death, or high hemostasis in the wattles and combs: infectious laryngotracheitis in chickens, duck plague, acute poisoning, acute poultry cholera (pasteurellosis), and other septicemic illnesses [9]. In addition to AI viruses, various respiratory illnesses and decreased egg production should be considered. These include bacterial infections, chlamydia, mycoplasma, IB, lentogenic ND virus, avian pneumovirus, and other paramyxoviruses.

## Clinical Symptoms

The type of AI virus and host species determines the clinical manifestations, severity, and fatality rates of AI. Most AI viruses are LPAI viruses (subtypes H1–H16) [50]. However, some AI H5 and H7 viruses are HPAI viruses and are very deadly to chickens, turkeys, and other domestic fowl that contain bile [81]. The majority of wild birds have subclinical AI virus infection [50]. The exception is the H5 HPAI virus of the Eurasian lineage. The Eurasian virus has been linked to deaths in domestic and wild ducks and other wild and domestic bird species. In certain cases, it has also been linked to significant deaths in wild bird species such as herons, turkeys, black vultures, and several types of pelicans [82].

An infection with the LPAI virus typically results in respiratory symptoms in birds, including sneezing, coughing, nasal and eye discharge, and swelling of the infraorbital sinuses [83]. Sinusitis is common in ducks, quails, and domestic turkey. Respiratory tract lesions typically involve inflammation and blockage of the lungs and trachea [84]. AI symptoms in laying and broiler hens include mucosal edema and inflammatory exudate in the oviduct lumen, decreased egg production, infertility, and egg rupture or involution [85]. Symptoms that are rarely observed in laying hens and broilers include acute renal failure and deposition of visceral uric acid (visceral gout) [86].

Clinical symptoms or severe AI-related lesions may not be visible in acute cases before death [9]. However, in severe cases, the lesions could be as follows: Cyanosis and edema of the head, comb, wattles, and snood (in turkey); ischemic necrosis of the comb, wattle, or hair net; edema and red discoloration of the calves and feet as a result of subcutaneous ecchymotic bleeding; petechial hemorrhages in the muscles and visceral organs; and blood staining [66]. Greenish diarrhea is common in severely sick birds [87]. Acute AI infection-surviving birds may develop central nervous system (CNS) involvement, which manifests as torticollis, incoordination, opisthotonos, paralysis, and drooping wings [88]. The location and severity of microscopic lesions vary widely; examples include edema, bleeding, and necrosis in the parenchymal cells of the skin, CNS, and various visceral organs [55].

The symptoms of AI virus infection can range from moderate to severe, especially in those infected with the H5N1 or H7N9 subtypes [89]. These symptoms, which include sore throat, stuffy nose, fever, cough, body aches, headache, exhaustion, and conjunctivitis, are comparable to seasonal flu [90]. Types A and B influenza viruses cause asymptomatic respiratory infections in young and healthy people, but in certain cases, especially in older patients and those with comorbidities or immunosuppressed illnesses, they can be fatal [91]. Symptoms include cough, malaise, fever, chills, sore throat, headache, coryza, anorexia, and myalgia [92]. There is a 3–4 day symptom period after the 1–4 days incubation period. The biological characteristics of the virus, the individual’s health before infection, and pre-existing immunity are some of the variables that affect the outcome of infection [93]. A more serious infection is more likely to occur when certain risk factors are present, such as immunological diseases, kidney failure, heart or lung disease, and smoking [94]. The influenza C virus typically does not cause signs of illness, but the H3N2 influenza virus is the most severe of the influenza viruses, followed by the influenza B virus and the influenza H1N1 virus [95, 96].

## Transmission

Human infection with AI viruses typically results from direct virus transmission from birds to humans [97]. AIV is usually transmitted by wild birds through water in an oral-fecal way, and such transmission may also be the cause of zoonotic human infections. The capacity to replicate in humans is known to exist for the AI virus subtypes H1N1, H3N2, H3N8, H4N8, H6N1, H6N2, H9N2, and H10N7 [98]. This replication was tested experimentally in 81 healthy people. Individuals infected with H4N8, H10N7, or H6N1 exhibit minor symptoms in the upper respiratory tract and nasopharyngeal virus replication.

Nevertheless, there was no success in spreading H6N1 from an infected individual to a healthy individual. There is no symptomatic indication of viral replication in the nasopharynx of individuals infected with H1N1, H3N2, H3N8, H6N2, or H9N2. These findings suggest that subtypes H1 or H3 and N1 or N2 offer cross-reactive immunity to stop avian virus replication [5]. Other avian viruses, such as H7N7, H9N2, and H7N3, can infect humans in addition to H5N1 [7]. This variety can infect terrestrial birds. Human influenza viruses bind to NeuAcα2.6Gal receptors found in land fowl, such as chickens and ducks [99]. This implies that both people and land bird species are potential hosts of the avian virus subtype H9N2.

The first human infection with the AI subtype H5N1 virus occurred in 1997 in Hong Kong, resulting in 18 cases and 6 of them resulted in death [34]. The poultry market, where ducks, geese, chickens, and other animals are sold for human consumption, is the source of this virus [16, 100]. An AI infection in the People’s Republic of China in February 2003 caused severe acute respiratory illness in a father and son [5]. There appears to be no evidence of NA protein stalk mutations. However, there is an amino acid replacement at position 227. This mutation allows the virus to bind to the avian sialic acid alpha (SA−α) 2.3 receptor and the human SA-α2.6 receptor, which does not alter the ability of the virus to cause human-to-human transmission [101].

Most AI cases in humans occur by handling or direct contact with infected birds and by killing or preparing infected birds for food [102]. In other situations, raw infected blood was the source of infection, although the birds did not exhibit symptoms [103]. The respiratory, digestive, and conjunctival tracts are the most likely points of entry for transmission from birds to humans [104]. Wild birds can spread the AI virus. The fact that the virus is spreading across regions where no previous cases of the virus have been found makes wild water birds more vulnerable because their migration patterns align with these regions [13]. The AI H5N1 virus was discovered in 2006 in some wild water birds in Western Europe, mainly in regions where the virus had not previously been reported [105]. The primary defense against using wild water birds as disease vectors is that if they contract the disease, the animals will either pass away from their illness too soon or become too ill to fly large distances and infect humans.

The AI virus can mostly continue replicating and subsequently multiply within chickens by keeping unsold live birds overnight in poultry markets [106]. Compared with the entering birds, these birds had higher levels of viral isolation. The virus can be prevented from spreading by implementing “rest days,” during which chicken markets are shut down entirely [107]. Although this virus can spread quickly in chicken markets, it can also spread efficiently to other poultry farms through empty cages [108]. A novel subtype of the AI H5N1 clade 2.3.4.4b HA virus was identified in April 2023 [109]. This new subtype did not exhibit any signs of genetic reassortment through antigenic shift; instead, it was found to be 99% similar to the old highly pathogenic H5N1 subtype. Most South American wild birds belong to this novel category [109].

## Risk Factor

Although human infections with AI viruses are rare, they have occasionally been documented. Humans may contract the infection through direct or indirect contact with infected animals [110]. There is no evidence that this AI virus can spread persistently from person to person. Exposure to diseased birds, either alive or dead, or polluted environments like poultry markets appears to be the primary risk factor for human transmission [111]. Other possible risk factors for disease spread include slaughter, butchering, handling contaminated poultry carcasses, and preparing poultry for eating, particularly at home [112]. There is no evidence that properly prepared and cooked chicken or eggs can spread the AI virus subtypes H5N1, H7N9, or any other subtype to people [113]. Multiple human cases of H5N1 AI are related to eating meals prepared with tainted raw chicken blood [114].

## Public Health Importance

AI disease outbreaks in domestic and wild birds are uncommon; however, an infection can significantly threaten public, veterinary, and medical health. Following the 1997 epidemic of AI H5N1 in people and poultry in Hong Kong and the 2003 outbreak of AI H7N7 in the Netherlands, there have been concerns that the AI virus may continue to exist in some chicken populations and, through multiple mutations or reassortment, become a pandemic virus for humans [34, 115]. A pandemic-causing AI virus must be able to spread from person to person, resulting in high rates of illness or death [116]. The viruses that emerged in the 20^th^ century were novel HA subtypes against which the human population failed to develop immunity [30]. The reintroduction of H1N1, which over time modified and re-sequenced the AI A genes of multiple AI, human, and swine viruses, resulted in the 2009 H1N1 pandemic [117]. The current strains of AI viruses that pose a threat to global health are many subtypes, namely H5, H7, and H9, which have repeatedly infected humans and caused occasional diseases [118]. One health approach to mitigating the AI virus is vaccinating humans and susceptible farmed and pet animals.

## Economic Impact

Most AI H5N1-affected nations report poultry losses of approximately 1% of the gross domestic product, with losses reaching as high as 0.6 in Vietnam [119]. This causes the virus to gradually spread to other avian hosts. More than 250 million birds were killed or left dead due to the disease in June 2007, which had an estimated financial impact of more than US$ 12 billion across 62 countries [105]. It has previously been suggested that information systems developed by academic, commercial, and research organizations can lessen the effects of this illness. Due to fighting many AI virus outbreaks between 1983 and 2005, 356.64 million hens died [120].

Threats of a worldwide pandemic have existed since the H5N1 strain of AI first appeared in Hong Kong in 1997. More than 200 million birds have been killed by the H5N1 AI subtype and its variants, costing the poultry industry more than US$ 10 billion in losses from research and human lives [121]. In China, business sales dropped by US$ 2.5 billion, while farmer losses approached US$ 1 billion in 2004 [122]. In 2005, Cambodia, Thailand, and Vietnam suffered losses of US$ 560 million [105].

Trade-related nations establish national laws and adopt international guidelines on animal health. To coordinate efforts to manage the disease, a timeline of events can be created from the start of the outbreak to the start of production. Losses in an affected area can provide benefits to unaffected areas [5]. Regionalization and long-term collaborations between public and commercial organizations can improve epidemiological research, epidemic models, service infrastructure, statistical and economic evaluations, and networks and information systems [13].

In 2004, at the peak of its epidemic, the likelihood of an AI virus infection in Thailand was assessed based on the species and type of virus. The highest risk animal is the quail (1.3%), which is followed by laying hens (0.25%) and broilers (0.25%), ducks and geese (0.075%), and free-range chickens (0.05%) as the least likely animals to contract this illness [123]. Thailand was the country most affected by the disease in January–April 2004, with a decline in exports of 75%, followed by China with a decline of 63%, Hong Kong at 55%, and the United States at 27%, while Brazil was the only country that increased exports by 6% [120].

Brazil profited from the bans placed between 2003 and 2005 on Asian nations that exported chicken, especially Thailand and Turkey. Brazil’s processed meat sales climbed from US$ 220 to US$ 398 million, while the country’s non-processed meat sales doubled from US$ 1.5 billion to US$ 2.9 billion [124]. In 2006, consumer markets for meat and eggs declined in most Asian countries; this was the case in 15% of non-affected countries such as Argentina and Brazil and 30% of disease-affected countries. In that year, poultry meat exports from China, Brazil, and the European Union decreased by 13%, 7%, and the European Union by 2%. In addition, in that year, the United States had a 39% global market share, followed by Brazil at 37%, the European Union at 12%, and China at 1.9%. Nonetheless, Brazil now receives 17% of the world’s exported cooked beef, up from 13% in 2005 [125].

## Treatment

AI, which affects poultry, has no known cure. However, secondary illnesses can be prevented using broad-spectrum antibiotics, sound husbandry practices, and a healthy diet [126]. Treatment for AI in humans varies among individuals based on the severity of the disorder. The illness may involve a range of drugs in addition to symptomatic care, such as antivirals and antibiotics, to treat or prevent subsequent bacterial pneumonia [127]. Certain AI viruses can be effectively treated with two classes of antiviral medications: adamantane (rimantadine and amantadine) and NA inhibitors (zanamivir, peramivir, laninamivir, and oseltamivir) [128]. However, some of these medications (laninamivir and peramivir) are not licensed in all countries.

The first antiviral medications to treat AI were rimantadine, amantadine, and adamantane [128]. This substance blocks the M2 ion channel, preventing the reproduction of AI viruses during the uncoating phase [129]. However, the rapid creation and spread of drug-resistant variants limits the efficacy of these antiviral agents. The crystal structure of the influenza NA complex, including sialic acid and the sialic acid derivative 2-deoxy-2,3-dehydro-N-acetyl-neuraminic acid, was determined, which led to the synthesis of neuraminidase (NA) inhibitors such as zanamivir and oseltamivir [130]. This inhibitor prevents the virus from escaping from infected cells and entering the respiratory system by blocking the active site of the NA enzyme [22]. Since all AI viruses appear to have a highly conserved enzymatic active site, these medications may offer protection against any subtype of AI virus that may develop in humans.

**Figure-1:**
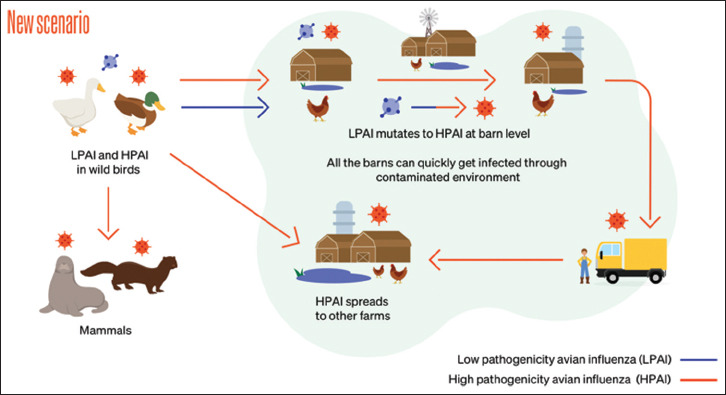
Transmission of highly pathogenic avian influenza [Source: https://www.woah.org/app/uploads/2023/06/avian-influenza-understanding-new-dynamics-to-better-combat-the-disease.pdf].

## Vaccination

Vaccinating poultry against AI disease using live recombinant vaccine (Fowlpox H5) and inactivated vaccine can limit virus transmission when vaccinated birds become sick, protect birds from clinical disease, and increase resistance to infection [131]. Therefore, by reducing the number of circulating viruses, carefully controlled poultry vaccination can reduce rates of mortality and morbidity, as well as human danger. The best public health measure to prevent influenza in people is annual influenza vaccination, which comes in two trivalent formulations: live attenuated and inactivated formulations containing AI A virus strains (H1N1 and H3N2) and AI B virus [132]. The WHO coordinates the semiannual strain selection procedure to establish the makeup of vaccines in the northern and southern hemispheres [45].

No vaccine for AI is commercially available and has undergone experimental testing that satisfies all the necessary criteria. Most vaccinations achieve the intended outcome, which is protection against clinical illness caused by AI viruses [133]. The main objective of control strategies, which aim to eradicate virulent field viruses, is to determine how well viral excretion can be reduced [134]. Therefore, producing vaccines quickly and effectively is the best action to stop the AI pandemic.

## Control

Risk management from farm to table is necessary for AI control in poultry in rural and commercial sectors. Some of these basic needs include putting good agricultural practices into practice, such as teaching workers about biosecurity and good management practices, particularly with regard to culling poultry; creating a biosafe environment to isolate poultry from potential carriers of the AI virus; providing a safe and contaminated feed supply; disinfecting and decontaminating equipment before introducing a new flock or following the culling of an existing flock; regularly composting manure and carcasses for all flocks; and safely disposing of carcasses from farms known to be infected [135].

Effective risk management requires open communication between employees, veterinarians, and animal suppliers [136]. Following an outbreak, vaccination campaigns, controlled depopulation, rapid eradication, and disease surveillance and inspection must be implemented [137]. Vaccination programs might vary by nation, but it is not a good idea to put off these control measures when depopulation is occurring quickly because doing so could cause massive financial losses [18]. In contrast, quick actions can drastically reduce costs and stop infection spread. As affected nations must approach biosecurity upgrades as a cost-effective investment, efficient risk communication with farmers and producers is essential at the national level [138]. Poultry infected with HPAI and its commercially manufactured products are not allowed to enter the food chain in developed nations. Furthermore, in industrialized nations, LPAI and HPAI viruses are uncommon in commercial and non-commercial poultry [139].

Local food safety practices in developing nations infected with the H5N1 subtype AI virus include keeping food cold, washing all surfaces, separating raw and cooked meat, and cooking meat to a proper temperature of 70°C [116]. Food manufacturers should be aware that the AI virus can withstand freezing and refrigeration. Refrigeration is not a control method because low temperatures stabilize the virus [140]. Thus, proper hygiene is crucial for preventing the spread of these diseases. Successful vaccination reduces the probability of AI H5N1 virus infection in meat and other poultry products and prevents systemic infection in chickens [141].

## Conclusion

AI has been recognized as one of the worst zoonotic diseases due to its grave public health and economic impact, especially with regard to poultry loss from deaths of birds. AI is not restricted to specific regions, as it has been reported in virtually all continents of the world. More challenging is the highly pathogenic nature of the influenza virus, which can cause serious infections in humans due to direct virus transmission from infected birds. Exposure to diseased birds, either alive or dead, or polluted poultry markets, slaughter, butchering, and handling contaminated poultry carcasses are widely reported primary risk factors for its zoonotic transmission to humans. Besides being isolated from poultry and humans, the influenza virus has also been isolated in pet animals, wild birds, and other wild animals. An infection with the Influenza virus typically results in respiratory symptoms such as swelling of the infraorbital sinuses in birds, sneezing, coughing, nasal discharge, and eye discharge. Other common symptoms in infected humans include sore throat, stuffy nose, fever, cough, chills, body aches, headache, exhaustion, and conjunctivitis. The AI virus is usually diagnosed in a laboratory using molecular or serological tests. Currently, there is no effective treatment for AI virus infections in commercial poultry; however, infections due to AI in humans are usually limited to supportive therapy and antiviral medication. More complicating is the frequent emergence of resistance by the influenza virus to currently used antivirals. However, a series of data and reports have shown that successful vaccination strategies within the One Health concept, such as the quick development of effective vaccines and good hygienic practices, will be very impactful in curtailing the recurring AI incidences and pandemics.
